# Pre and post-competitive anxiety and self-confidence and their relationship with technical-tactical performance in high-level men's padel players

**DOI:** 10.3389/fspor.2024.1393980

**Published:** 2024-06-10

**Authors:** Rafael Conde-Ripoll, Adrián Escudero-Tena, Álvaro Bustamante-Sánchez

**Affiliations:** Faculty of Sport Sciences, Universidad Europea de Madrid, Madrid, Spain

**Keywords:** psychology, match analysis, racket sports, CSAI-2R, STAI-S

## Abstract

**Introduction:**

This study aimed to analyze the variations in pre- and post-competitive anxiety and self-confidence considering match outcomes, the performance according to the result and the correlation among performance and psychological variables.

**Methods:**

The sample consisted on 12 matches in which 11 high-level padel players from Finland (top 24) voluntarily participated. CSAI-2R and STAI-S were used to assess psychological variables and technical-tactical performance was evaluated by a certified padel coach.

**Results:**

Losing players presented higher cognitive (*p* = .004), somatic (*p* = .020) and state (*p* = .001) anxiety and lower selfconfidence (*p* = .014), and winning players showed higher state anxiety (*p* = .022), after than before the matches. Post-match, winning players exhibited higher self-confidence (*p* = .015) than losing players. Winning players made more winners (*p* = .010) than losing players. There are direct correlations between unforced errors and post-state anxiety (*p* = .015), unforced errors and state anxiety (*p* = .009) and winners and cognitive anxiety (*p* = .033), in winning players; between generators of forced error and post-cognitive anxiety (*p* = .034), forced errors and cognitive anxiety in losing players (*p* = .001). There are indirect correlations between forced errors and pre-cognitive anxiety (*p* = .009), winners and somatic anxiety (*p* = .046), unforced errors and state anxiety (*p* = .042) in losing players.

**Discussion:**

The outcomes imply the need for intervention programs to equip players, especially those facing defeat, with tools for anxiety management and self-confidence enhancement. Likewise, coaches are advised to incorporate exercises that promote the occurrence of winners.

## Introduction

The sport of padel is witnessing exponential growth worldwide, with participation from more than 80 nations affiliated with the International Padel Federation (1). This expansion has prompted a marked rise in scholarly investigations, particularly focused on assessing technical and tactical performance (2–4).

Mental toughness, as evidenced by its capacity to enable athletes to uphold or enhance performance during challenging circumstances (5), holds a particular significance in the context of sports psychology. Within this domain, anxiety is seen as a concept characterized by an emotional reaction to a perceived threat, blending physiological arousal and cognitive apprehensions (6). In a competitive scenario, a distinction can be made between a stable personality disposition termed trait anxiety (7), and the transient symptoms encountered during a specific competition, referred to as state anxiety (8). Cognitive anxiety encompasses adverse anticipations of success or self-evaluation, negative thinking, reduced self-worth, pessimistic inner dialogues, fear of failure, diminished self-belief, performance worries, visions of failure, challenges in concentration, and disrupted attention (9–11). Somatic anxiety is linked to autonomic arousal, presented as increased heart rate or muscle tension, contributing to negative sensations like nervousness, breathing difficulties, heightened blood pressure, dry throat, muscle strain, rapid heartbeat, clammy palms, and a sensation of butterflies in the stomach (9, 11, 12). Self-confidence, defined as a player's conviction in his/her ability to perform effectively in competition (13), is a facet studied within this construct to gauge the athlete's comprehensive sense of accomplishment (14).

In this sense, competitive anxiety and self-confidence, specially prior to competition, has been extensively studied in recent decades (15–17). Research has shown that athletes with elevated anxiety levels often demonstrate poorer performance in competitions in comparison to those with lower anxiety levels (18–20). Additionally, there exists a direct correlation between the player's self-confidence and his/her performance in sports (20, 21). Once the competition is over, athletes from different sports such as football, basketball and volleyball show lower levels of competitive anxiety than moments prior to the event (22–24). The same happened to taekwondo athletes, who also showed higher levels of self-confidence at this stage (25). However, to the best of our knowledge, there is only one study, in tennis, which distinguishes winning vs. losing players regarding post-competitive anxiety and self-confidence, with the former showing notably reduced cognitive anxiety and elevated self-confidence compared to the latter (16).

A key performance indicator shaping match results in professional padel is the effectiveness of the ultimate shot (26–28). From these investigations, it has been established that a point in the game can culminate through a winner, a forced error or an unforced error. A winner occurs when a player secures the point directly (i.e., after the ball bounces correctly on the opposing side after crossing the net, the ball bounces for a second time; or the ball strikes the opponent's body before being out). Conversely, a forced error happens when a player loses the points due to difficulty in executing a shot or being poorly positioned following the opponent's shot. Finally, an unforced error arises when a player loses the point despite facing a relatively straightforward situation with favorable space-time conditions (27). Winning pairs commit more winners and less unforced errors than losing pairs (2, 29, 30). Nevertheless, while previous articles dissect each method of concluding the point separately, our research not only does so but also advocates for the adoption of technical-tactical performance ratios. These ratios are advantageous as they remain unaffected by the number of points, games, or sets. Moreover, regarding the court area, winners are mainly executed from the net area (30) while errors, regardless of the type, are evenly distributed between the net and the back of the court. In absolute terms, overheads and volleys are the primary sources of winners (31), whereas forced errors often result from volleys, and unforced errors mainly arise from volleys and groundstrokes with no wall (26).

Upon reviewing the scientific literature, the combination of mental preparation and performance in padel has received limited attention, with only a few studies exploring precompetitive anxiety and self-confidence (32–34) and none of them studying post-competitive anxiety and self-confidence. Understanding the interplay between a player's anxiety, self-confidence, and performance holds substantial value for players, coaches, and sports psychologists. This insight can enable players to adapt their playing style, while coaches and psychologists can customize feedback and training sessions accordingly. The aims of the present investigation were to analyze: (1) the differences between pre and post-competitive anxiety and self-confidence during matches in high level men's padel players from Finland as a function of the result*,* (2) the differences between winning and losing players regarding anxiety and self-confidence as a function of the moment (pre or post-match)*,* (3) the technical-tactical performance according to the result and (4) the correlation among technical-tactical performance, anxiety and self-confidence. Therefore, the following hypotheses were put forward: (1) match winning players will show higher levels of self-confidence and lower levels of anxiety in the post-match compared to pre-match; the opposite will happen in the match losing players, (2) before and after the match, respectively, match winning players will display higher levels of self-confidence and lower levels of anxiety than match losing players, (3) match winning players will make more winners and generators of forced errors, and less forced and unforced errors than match losing players, (4) there will be a direct correlation between winners and self-confidence in match winning players, whereas in match losing players, there will be a direct correlation between errors and anxiety, and an indirect correlation between errors and self-confidence.

## Material and methods

### Study design

The design of this research is framed under an empirical methodology and more specifically it is a study with a descriptive strategy. On one hand, questionnaires are applied (psychological variables) (35) and, on the other hand, matches are observed, the latter being nomothetic, punctual and multidimensional (technical-tactical performance variables) (36).

### Sample and participants

**Figure 1:**
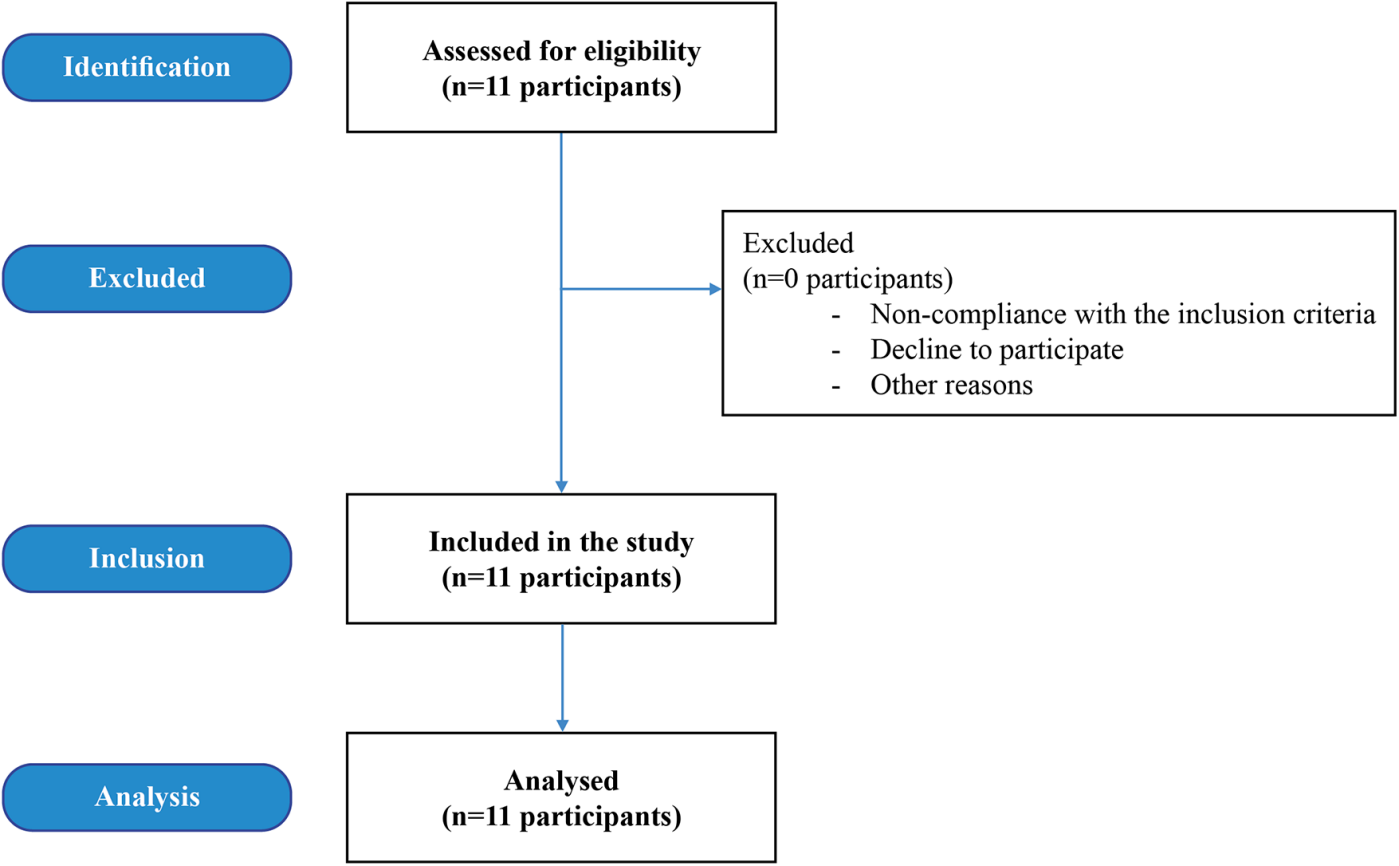
STROBE flowchart. Adapted from https://www.strobe-statement.org/.

We analyzed 12 matches in which 1,514 points were disputed. Following a convenience sampling, these matches were played by a total of 11 men's high-level padel players (27.91 (5.03) years old and 4.64 (1.86) years of competitive experience) from Finland who voluntarily participated in the present study. This represents almost 50% of the total target population. An observational study of elite athletes from volleyball included 14 athletes who were in preparation for competing in important events, representing a similar sample size in a similar context (high-level athletes prior to competition) (37). The STROBE flow chart (Figure 1) was used to ensure that the clear assessment of participants (38). All participants were ranked top 24 in Finland. None of the athletes had any physical injuries nor were they taking any medication at the time of the measurements. In addition, none of the participants had any reason that prevented them from participating in the study.

The study was in accordance with the Helsinki Declaration (39). Participants were treated ethically under the American Psychological Association code of ethics regarding consent, anonymity and responses. Previously, the current investigation had been approved by the Ethics Committee of the European University of Madrid with the code CIPI/22.303. So as to respect the principles of voluntariness and confidentiality, each player was required to sign an informed consent form that clearly explained the objectives of the research and their voluntary participation in it.

### Study variables

To carry out this study, the following variables were taken into account:

- Psychological variables: somatic anxiety, cognitive anxiety, self-confidence (9, 11, 13) and state anxiety (40). CSAI-2R questionnaire was used to measure somatic and cognitive anxiety and self-confidence of players (41) and STAI-S questionnaire was used to measure their state anxiety (42). These questionnaires have been used in previous research in padel (34). All questionnaires were completed in a quiet room with controlled temperature of 20°C. Participants completed the questionnaires in English, as it is the only language that both researchers and athletes are fluent in. Participants were not allowed to speak during the assessments. In the analysis of the CSAI-2R instrument, Cronbach's alpha coefficients were obtained, showing reliability scores of .75 (for pre), .72 (for post) for cognitive anxiety, .84 (for pre), .90 (for post) for somatic anxiety, and .72 (for pre), .88 (for post) for self-confidence, all meeting acceptable standards (43–45).
- Technical-tactical performance variables [defined based on their categorical core and degree of openness (46)]:
- Effectiveness of the last shot: a difference was made between winner, forced error and unforced error (27).
- Forced error generator: shot which induces a forced error in the opposing pair (47).

In addition, technical-tactical performance ratios were calculated using the above mentioned technical-tactical performance variables. It is worth noting that the effectiveness of the shots is considered a performance indicator in racket sports (48).

### Procedure

The questionnaires were administered to the players between 30 and 45 min prior to the start of each practice match, following the same criteria to that used by Conde-Ripoll et al. (34). 15–30 min after the practice match is over, the questionnaires were administered for post-competitive anxiety and self-confidence.

During each practice match, which followed the International Padel Federation rules, a certified padel coach with more than 10 years of experience, recorded the technical-tactical performance study variables through an ad-hoc instrument in Excel, following similar criteria than previous research (49, 50). At the end of the collection process, an intra-observer reliability analysis was performed to ensure the veracity of the data collected. The observer reanalyzed a random sample of 3 matches (matches were previously recorded) to ensure enough relevant data to represent 10%–20% of the study sample (51). The mean intra-observer reliability was.90, considered almost perfect (52). In addition, another observer, a doctor in sports sciences, with more than 20 JCR scientific articles published related to the topic of study, also analyzed a random sample of 3 matches to calculate the average inter-observer reliability, which was.84 (52).

### Statistical analysis

Shapiro-Wilk (considering *n* = 11 athletes) and a Kolgomorov-Smirnov (considering *n* = 1,514 points) tests were used to test the normality of the distribution of the data and it indicated that it is non-parametric. Then, a descriptive analysis was performed to obtain information on the number of times each study variable occurred (median and interquartile range).

Next, inferential analyzes were then conducted, including Wilcoxon signed-rank and Mann-Whitney′s *U*-tests. Additionally, effect sizes [*r*] were calculated for the non-parametric tests, which were classified as follows: .5 is a large effect, .3 is a medium effect and .1 is a small effect (53, 54).

Finally, a bivariate correlation analysis among psychological and technical-tactical performance variables in different contexts using Spearman's correlation.

All data were analyzed using the statistical package SPSS for Macintosh v.25.0 (SPSS Inc, Chicago, IL, United States) and a *p* value of less than .05 was considered to be statistically significant.

## Results

As can be seen in Table 1, regarding differences between before and after the matches, losing players showed a significant surge in cognitive, somatic and state anxiety alongside a significant decline in self-confidence; whereas winning players only displayed a significant increase in state anxiety. Besides, match-winning players significantly exhibited more self-confidence post-match compared to losers.

**Table 1:** Anxiety and self-confidence according to the moment (pre and post) and match outcome.

Match outcome	Variable	Pre median (IQR)	Post median (IQR)	Pre vs. post-measure		Winning vs. losing players pre		Winning vs. losing players post		Winning vs. losing players pre-post	
				*p*	ES	*p*	ES	*p*	ES	*p*	ES
Winning player	CA	1.50 (.60)	1.60 (75)	.407	.169	.361	.132	.457	.107	.064	.267
Losing player		1.30 (.40)	1.70 (.60)	.004*	.592						
Winning player	SA	1.29 (.43)	1.43 (.29)	.731	.070	.328	.141	.323	.143	.124	.222
Losing player		1.43 (.54)	1.50 (1.32)	.020*	.475						
Winning player	SC	3.20 (.60)	3.20 (.60)	.746	.066	.123	.222	.015*	.353	.093	.242
Losing player		3.00 (.40)	3.00 (.80)	.014*	.503						
Winning player	STA	5.50 (3.00)	6.00 (5.00)	.022*	.466	.338	.138	.112	.229	.192	.188
Losing player		6.00 (5.00)	8.50 (7.00)	.001*	.685						

CA, cognitive anxiety; SA, somatic anxiety; SC, self-confidence; STA, state anxiety; *p*, *p*-value; ES, effect size.

* *p* < .05.

Evidenced in Table 2, winning players significantly produced more winners than losing players per match. The same occurred in every single technical-tactical performance ratio [for example: winner/unforced error or (winner plus generator of forced error)/unforced error].

**Table 2:** Differences in technical-tactical performance of the padel players according to the match outcome.

Variable	Winning players		Losing players		*p*	ES
	Median	IQR	Median	IQR		
W	13.50	7.00	9.00	6.00	.010*	.373
GFE	8.00	4.00	7.50	5.00	.298	.150
FE	7.00	5.00	9.00	4.00	.110	.231
UE	9.50	5.00	12.00	6.00	.356	.133
W/UE	1.31	0.82	0.91	0.55	.001*	.460
(W + GFE)/UE	2.10	1.22	1.63	0.95	.002*	.442
W/(FE + UE)	0.85	0.51	0.49	0.25	.001*	.502
(W + GFE)/(FE + UE)	1.33	0.75	0.83	0.36	<.001*	.515

W, winner; GFE, generator of forced error; FE, forced error; UE, unforced error; SD, standard deviation; *p*, *p*-value; ES, effect size.

* *p* < .05.

As depicted in Table 3, there are indirect correlations between somatic and cognitive anxiety, between state anxiety and self-confidence, in winning players; and between forced errors and cognitive anxiety in losing players.

**Table 3:** Correlations between the pre-competitive values of the psychological and technical-tactical performance variables, in winning and losing players.

	Winning players							
	CA	SA	SC	STA	W	GFE	FE	UE
CA	1	−.558**	−.094	.056	−.211	.029	.104	.295
SA		1	−.281	.456*	.090	.194	−.035	−.217
SC			1	−.662**	.146	.063	−.332	.019
STA				1	−.259	−.018	.299	−.097
	Losing players							
	CA	SA	SC	STA	W	GFE	FE	UE
CA	1	.377	−.305	.458*	.066	.225	−.524**	.247
SA		1	.022	.500*	.111	−.057	.181	.160
SC			1	−.186	.356	−.207	.080	.136
STA				1	−.033	.236	.070	−.049

CA, cognitive anxiety; SA, somatic anxiety; SC, self-confidence; STA, state anxiety; W, winner; GFE, generator of forced error; FE, forced error; UE, unforced error.

* *p* < .05.

** *p* < .01.

In addition, there are direct correlations between state anxiety and somatic anxiety in winning players; between state anxiety and cognitive anxiety, and between state anxiety and somatic anxiety, in losing players.

As shown in Table 4, there are indirect correlations between state anxiety and self-confidence in winning players; and between self-confidence and cognitive anxiety, between self-confidence and somatic anxiety, between state anxiety and self-confidence, in losing players.

**Table 4:** Correlations between the post-competitive values of the psychological variables, and technical-tactical performance variables, in winning and losing players.

	Winning players							
	CA	SA	SC	STA	W	GFE	FE	UE
CA	1	−.221	−.054	.621**	.098	.077	.020	.299
SA		1	−.361	.082	.153	.147	−.085	−.122
SC			1	−.533**	.126	−.025	−.172	−.201
STA				1	.197	.094	.316	.490*
	Losing players							
	CA	SA	SC	STA	W	GFE	FE	UE
CA	1	.602**	−.586**	.285	−.146	.433*	.180	−.131
SA		1	−.412*	.619**	−.233	.089	.189	−.167
SC			1	−.444*	.240	−.247	−.203	.049
STA				1	−.313	.182	.227	−.299

CA, cognitive anxiety; SA, somatic anxiety; SC, self-confidence; STA, state anxiety; W, winner; GFE, generator of forced error; FE, forced error; UE, unforced error.

* *p* < .05.

** *p* < .01.

In addition, there are direct correlations between state anxiety and cognitive anxiety, between unforced errors and state anxiety, in winning players; and between somatic anxiety and cognitive anxiety, between state anxiety and somatic anxiety, between generators of forced error and cognitive anxiety, in losing players.

Highlighted in Table 5, there are indirect correlations between self-confidence and somatic anxiety in winning players; and between winners and somatic anxiety, between unforced errors and state anxiety in losing players.

**Table 5:** Correlations between values of the psychological (post minus pre) and technical-tactical performance variables in match winning and losing players.

	Winning players							
	CA	SA	SC	STA	W	GFE	FE	UE
CA	1	.502*	−.177	.296	.435*	.097	−.077	.155
SA		1	−.592**	.499*	.078	−.129	−.049	.248
SC			1	−.350	.042	−.093	.188	−.154
STA				1	.336	.099	.157	.520**
	Losing players							
	CA	SA	SC	STA	W	GFE	FE	UE
CA	1	.483*	−.377	.492*	−.195	.274	.643**	−.402
SA		1	−.314	.264	−.410*	.106	.162	−.384
SC			1	−.254	−.007	−.155	−.256	−.076
STA				1	−.382	.047	.240	−.418*

CA, cognitive anxiety; SA, somatic anxiety; SC, self-confidence; STA, state anxiety; W, winner; GFE, generator of forced error; FE, forced error; UE, unforced error.

* *p* < .05.

** *p* < .01.

In addition, there are direct correlations between somatic anxiety and cognitive anxiety, state anxiety and somatic anxiety, winners and cognitive anxiety, unforced errors and state anxiety in winning players; and between somatic anxiety and cognitive anxiety, between state anxiety and cognitive anxiety, between forced errors and cognitive anxiety in losing players.

Illustrated in Table 6, there are direct correlations between unforced errors and generators of forced error, and between unforced errors and forced errors, in winning players; and between unforced errors and winners in losing players.

**Table 6:** Correlations between technical-tactical performance variables, in match winning and losing players.

	Winning players			
	Winner shot	Generator of forced error	Forced error	Unforced error
Winner shot	1	.090	.042	.318
Generator of forced error		1	.245	.432*
Forced error			1	.511*
Unforced error				1
	Losing players			
	Winner shot	Generator of forced error	Forced error	Unforced error
Winner shot	1	.329	.246	.512*
Generator of forced error		1	.251	.119
Forced error			1	−.020
Unforced error				1

* *p* < .05

## Discussion

Our initial hypothesis suggested that winning pairs would exhibit higher self-confidence and lower anxiety in the post-match, compared to pre-match; while losing players would show the opposite pattern. Our findings partially supported this hypothesis. Winning players displayed higher state anxiety post-match, possibly due to unmet performance expectations or individual variability within winning pairs. Conversely, losing players demonstrated expected increases in anxiety and decreases in self-confidence post-match, potentially due to underperformance and emotional distress. This contrasts with prior research on singles tennis players on official competition (16). Overall, these findings highlight the contrasting impact of victory and defeat, indicating that while winning might not necessarily yield favorable effects, the repercussions of losing could be considerably detrimental to players. Consequently, coaches and players are prompted to participate in psychological training to skillfully manage the outcomes of triumph and loss (55), facilitating their continual evolution and growth as athletes. Additionally, recognizing padel as a partner sport emphasizes the interdependence between players, where individual performance directly affects the overall outcome, underscoring the importance of effective communication, trust, and mutual support within pairs.

The second hypothesis that match-winning players would exhibit higher self-confidence and lower anxiety before and after the practice match, compared to match-losing players, was partially supported. Prior to the match, no significant differences were found, consistent with previous research (34). Post-match, only self-confidence showed significant differences, with match-winning players exhibiting higher levels. This could be due to the fact that the increase of self-confidence is linked, among other factors, to positive performance results (56). In this line, Fuentes-García et al. (16) observed higher post-match self-confidence among winning elite junior tennis players compared to losers.

Additionally, it was third hypothesized that match-winning players would make more winners and forced error generators, and fewer forced and unforced errors compared to match-losing players. This hypothesis was partially supported. Significant differences were found in the number of winners, aligning with prior research (29, 30), highlighting the importance of winners as a key performance indicator in padel. Athletes should collaborate closely with their coaches to enhance strategies for increasing winners during training sessions. This could involve reviewing competitive or practice matches to discern winner shots and their underlying reasons (57). Additionally, coaches could implement exercises that emphasize and incentivize winners (58, 59). Although not statistically significant, winning players produced more generators of forced error, while losing players committed more forced and unforced errors. Regarding the latter, losing players significantly commit more unforced errors than winning players (2, 60) in the professional level. Additionally, winning players demonstrated technical-tactical performance ratios, emphasizing the usefulness of such ratios for evaluating padel players, as they are independent of the number of points, games, or sets. To illustrate, making 10 winners in a 6/0 6/0 match where each game is won at 40/0 (48 points in total) is not equivalent to achieving the same feat in a 7/6(5)—6/7(5)—7/6(5) match where each game is won at a golden point and each tiebreak was won 7/5 (120 points in total).

As a final hypothesis, it was established that there would be a direct correlation between winners and self-confidence in winning players, whereas in losing players, there would be a direct correlation between errors and anxiety, and an indirect correlation between errors and self-confidence. Acceptance of this hypothesis was partial. Winning players showed direct correlations between the number of winners and cognitive anxiety. This suggests that executing winners may evoke a heightened level of mental engagement, reflecting the complexity of the task. Likewise, significant direct correlations were found between generators of forced error and post-cognitive anxiety in losing players. This could be due to the defensive skills of the opponents, the winning players, who actively tried to reach even the most challenging shots. Significant direct correlations were found between unforced errors and state and post-state anxiety in winning players. Committing unforced errors may contribute to heightened state anxiety among winning players, since they may not like the idea of giving “free” points to the opponents through avoidable errors. And heightened anxiety levels indicate a decrease in sports performance (61, 62) and anticipation efficiency (63). Hence, these players could benefit from working closely with a sport psychologist, to develop resilience strategies to effectively cope with the frustration of pressure stemming from errors (64), emphasizing techniques such as reframing perspectives on mistakes. Significant direct correlations were found between forced errors and cognitive anxiety in the losing players. The rationale behind this observation might be losing players' emotional reactions to their failure in executing demanding shots, potentially influencing their perception of the effective technical-tactical maneuvers executed by winning players. Hence, coaches and sport psychologists could implement targeted training sessions focusing on stress management techniques and simulated match scenarios that replicate high-pressure situations (65). Indirect correlations were found between forced errors and pre-cognitive anxiety, and between unforced errors and state anxiety, among the losing players. When losing players make mistakes that they feel they can control and improve upon during practice matches, they may feel more responsible for those mistakes. Tailored approaches may assist in reframing perceptions of errors (66, 67) and pre-competition nervousness, ultimately aiding players in better managing their anxieties during matches. Furthermore, indirect correlations were found between winners and somatic anxiety among losing players. In fact, anxiety has been shown to impair sports performance (68–70).

### Practical applications

It is important to consider these results when structuring personalized training programs for each athlete and formulating task-specific training exercises. For example, doing exercises which make the athlete feel a higher level of self-confidence (71) and a lower level of anxiety after losing a practice match, especially if there is a tournament in the upcoming days may be of great importance for the future performance in that competition. Furthermore, coaches are instigated to integrate pressure training into their sessions with athletes to enhance athletes' performance in competition (65). This approach involves exposing athletes to pressure scenarios during practice, fostering their ability to perform proficiently under pressure (59), which can be defined as the athlete's heightened sense of the importance of performing well (72). It is essential to highlight that in padel, players are consistently required to make rapid decisions within brief timeframes (73) and the capability to manage pressure situations directly affects one's performance (74–76). Thus, coaches can implement consequences (such as judgement, forfeits, rewards), establish demands, and planned disruption during pressure training (77, 78). In this line, research indicates that consequences induce higher levels of pressure compared to demands (77), and an example of a consequence could be the head coach monitoring players' padel technical-tactical performance.

### Strengths

This study presents several strengths. Firstly, it pioneers research in padel by delving into pre- and post-competitive anxiety alongside self-confidence. Secondly, it stands as the initial study in padel to establish a correlation between technical-tactical performance and anxiety/self-confidence. Thirdly, its findings hold substantial practical implications for coaches and sports psychologists, particularly considering the differences in psychological variables in practice match losing players.

### Limitations and future studies

Despite employing a similar methodology to recent research in the domain, it is essential to underscore certain inherent limitations within this study. In future studies, researchers are encouraged to examine whether anxiety and self-confidence responses manifest similarly in both sexes. Although the questionnaires employed are valid and reliable, one specific limitation is that self-confidence and anxiety are assessed through self-perception, and some measurements of internal load (hormones such as cortisol, autonomic modulation or cortical arousal) could enhance the comprehension of the relationship between technical-tactical performance and anxiety/s`elf-confidence. Future research should consider athletes from different levels (beginners, amateur, professional players…).

## Conclusion

Losing players demonstrated elevated cognitive, somatic, and state anxiety, along with reduced self-confidence, while winning players experienced an increase in state anxiety post-match compared to their pre-match levels. Post-match, self-confidence was higher among winning players. In terms of technical-tactical performance variables, winning players made more winners than losing players. They also outperformed the losing players in every technical-tactical performance ratio. Besides, direct correlations were observed between unforced errors and state anxiety (both post-, and pre-post), and between winners and cognitive anxiety (pre-post) in winning players; between generator of forced errors and post-cognitive anxiety, and between forced errors and cognitive anxiety (pre-post) in losing players. Indirect correlations were observed between forced errors and pre-cognitive anxiety, between winners and somatic anxiety (pre-post), between unforced errors and state anxiety (pre-post) in losing players. Players are encouraged to develop their mental skills to manage errors and defeat. Likewise, coaches are encouraged to include pressure training and promote the appearance of winners in simulated matches.

## Data Availability

The raw data supporting the conclusions of this article will be made available by the authors, without undue reservation.
